# Controlled Moderate Interfacial Reaction Facilitates High‐Strength Low‐Resistivity Barrier Layer for Half‐Heusler Thermoelectrics

**DOI:** 10.1002/advs.74870

**Published:** 2026-03-17

**Authors:** Jian Liang, Shuyue Tan, Lifeng Jiang, Wen Zhang, Hongda Song, Xinghui Wang, Haiping Guo, Huijun Kang, Rongchun Chen, Enyu Guo, Zongning Chen, Tongmin Wang

**Affiliations:** ^1^ Key Laboratory of Solidification Control and Digital Preparation Technology (Liaoning Province) School of Materials Science and Engineering Dalian University of Technology Dalian China; ^2^ Ningbo Institute of Dalian University of Technology Ningbo China

**Keywords:** barrier layer, bonding strength, contact resistivity, Half‐Heusler, thermoelectric devices

## Abstract

Half‐Heusler compounds serve as a compelling solution for medium‐ and high‐temperature waste heat recovery owing to their exceptional thermal stability and superior thermoelectric properties. However, the practical application of their corresponding thermoelectric devices has long been constrained by thermal stress‐induced cracking, particularly at the interface between electrodes and thermoelectric material. Here, we engineer an atomically bonded interface via a well‐controlled interfacial reaction and propose a novel barrier layer design paradigm based on the thermodynamic parameters. By screening thermodynamic parameters, we have successfully fabricated a ZrNiSn/Co_25_Fe_50_Ni_25_ thermoelectric junction exhibiting high bonding strength (77.0 MPa), low contact resistivity (1.16 µΩ·cm^2^), and excellent thermal stability at 923 K. The corresponding single‐leg device achieves a power density exceeding 6.78 W/cm^2^ and a conversion efficiency of over 6.13%. After annealing at 923 K for 168 h, the junction maintains a bonding strength of 60.0 MPa and a contact resistivity less than 5 µΩ·cm^2^. These results underscore the pivotal role of controlled interfacial reactions in advancing medium‐ and high‐temperature half‐Heusler thermoelectric devices, providing a solid foundation for waste heat recovery applications.

## Graphical abstract

The barrier layer is designed comprehensively by thermal expansion coefficient and interfacial reaction energy to yield a controlled interfacial reaction. Ultimately, the Co_25_Fe_50_Ni_25_/half‐Heusler joint simultaneously achieves low contact resistivity and high bonding strength.

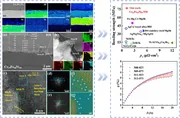

## 1 Introduction

Thermoelectric (TE) devices can directly convert waste heat into electrical energy via the Seebeck effect [1, 2, 3, 4, 5, 6, 7], thereby facilitating efficient recovery and utilization of medium‐to‐high temperature waste heat in industries such as metallurgy and chemical engineering, thereby contributing to sustainable energy development [8, 9, 10]. Among various TE material systems [11, 12, 13, 14, 15, 16, 17, 18, 19, 20], half‐Heusler (HH) alloys have emerged as a research focus for contemporary medium‐to‐high temperature TEs [21, 22, 23, 24]. Their superior high‐temperature thermal stability (enabling long‐term operation at 700–1000 K), moderate thermoelectric figure of merit (*zT* value generally exceeding 1), and compositional versatility make them prime candidates for commercial medium‐to‐high temperature TE devices [25, 26]. However, at elevated temperatures, metal electrodes tend to diffuse into TE materials, resulting in irreversible performance degradation. Therefore, barrier layer materials, which impede electrode diffusion, represent a core technical bottleneck for the commercialization of HH TE devices.

The barrier layer in TE devices serves as a critical hub for electron transport [27, 28, 29, 30], heat conduction, and mechanical support, directly dictating the actual output power, conversion efficiency, and service life of the devices [31, 32, 33]. An optimal barrier layer must exhibit high bonding strength with TE materials, low contact resistivity, and superior thermal stability. Verma et al. [34] fabricated a Ti/Ti_0.7_Nb_0.3_CoSb joint with excellent thermal expansion matching and atomic bonding, but encountered extremely high interface resistance (300 mΩ) due to an unsuitable diffusion layer and high barrier height. Simultaneously, the coefficient of thermal expansion (CTE) mismatch between the TE material and the barrier layer generates significant thermal stress during thermal cycling, triggering mechanical failures such as interfacial cracking and delamination. Sun et al. [35] have prepared a YbMg_0.8_Zn_1.2_Sb_2_/Fe joint where CTE incompatibility led to holes and cracks during annealing, causing bonding strength to drop from over 25 MPa to below 17 MPa and eventual device failure. To address these interfacial issues, researchers have explored various interfacial regulation strategies, such as using refractory metals (e.g., W and Mo) as barrier layers [33, 36] or optimizing interfacial bonding via magnetron sputtering [37]. However, the high electrical resistivity of refractory metals compromises the device output performance, while conventional coating technologies struggle to achieve atomic‐level bonding, resulting in insufficient high‐temperature stability [37]. Essentially, the core challenge in current interfacial design lies in balancing “interfacial reaction control” and “performance optimization”. Excessively suppressed reactions lead to insufficient bonding strength, while uncontrolled reactions promote detrimental phase formation.

**FIGURE 1:**
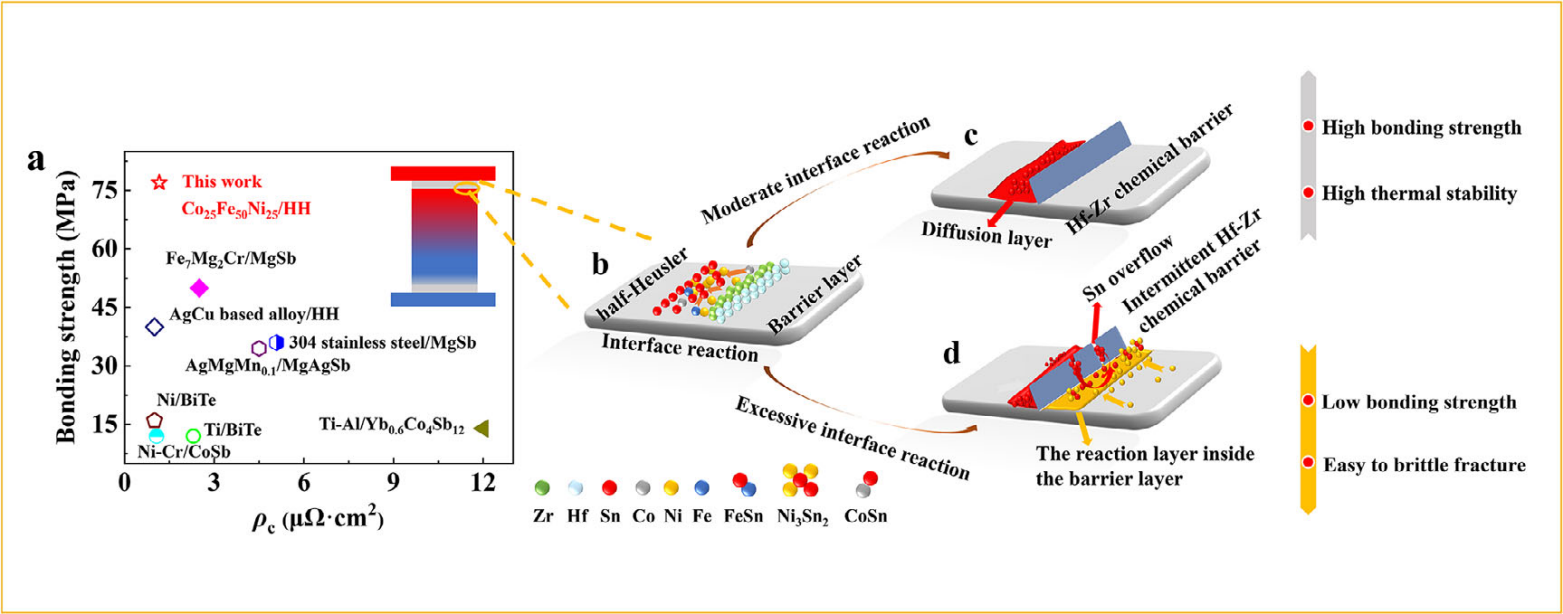
Mechanism of interface reaction: (a) comparison of contact resistance and shear strength in this study with other research results [38, 39, 40, 41, 42, 43, 44, 45], (b) atomic schematic diagram of interface reaction during sintering, (c) moderate interface reaction, and (d) excessive interface reaction.

Herein, we proposed a design strategy anchored in “controllable mild interfacial reactions”. As illustrated in Figure 1, we first screened elements based on the interfacial reaction energy (E*_IR_*) criterion, selecting Ni (the strongest binding energy) and the most reactive Sn within the Hf_0.6_Zr_0.4_NiSn_0.99_Sb_0.01_ matrix to guarantee enhanced bonding strength. Subsequently, Fe and Co were chosen based on CTE matching and low E*_IR_*, ultimately forming a cobalt‐iron‐nickel (CoFeNi) alloy. By minimizing cobalt content and adjusting the Ni/Fe ratio (Figure 1b), we controlled the extent of interfacial reactions. A dense and continuous Hf‐Zr chemical barrier layer effectively inhibited the Sn diffusion into the barrier layer, preserving joint performance and ensuring interfacial thermal stability (Figure 1c). On the contrary, excessive interfacial reaction disrupted the Hf‐Zr chemical barrier, allowing Sn diffusion into the barrier layer (Figure 1d). When the Hf‐Zr layer is continuously covered, and the thickness of the diffusion layer is less than 5 µm, the resulting joint exhibited a bonding strength of 77 MPa and a contact resistivity of 1.16 µΩ·cm^2^ (Figure 1a). The corresponding single‐leg device achieved a high conversion efficiency of over 6.13% at a temperature difference of 660 K. This work, guided by thermodynamic design criteria, provided theoretical support and technical guidance for fabricating high‐performance, long‐service‐life HH devices, advancing their practical applications in waste heat recovery.

## 2 Experimental Details

### 2.1 TE Materials and Barrier Layer Synthesis

High‐purity sponge‐shaped particles, including Hf (99.99%), Zr (99.99%), Ni (99.99%), Sn (99.99%), and Sb (99.99%), were employed for fabricating HH TE materials. Following the stoichiometric ratio of Hf_0.6_Zr_0.4_NiSn_0.99_Sb_0.01_, melting was performed in a magnetic levitation melting furnace (LMF, FM‐40, China). The alloy was melted four times, with the ingot flipped mechanically after each melt. The crushed ingot was then wet‐ball‐milled in alcohol using a planetary ball mill (Pulverisette 4, Fritsch, Germany) for 10 h. The resulting nanopowder was sintered via spark plasma sintering (SPS, SPS‐30, Chen Hua, China) at 1173 K under 65 MPa axial pressure for 10 min. Sintered wafers were cut into required dimensions using a diamond wire cutting machine: Ø12.7 mm × 1 mm for thermal conductivity measurements and 2 mm × 2 mm × 12 mm for Seebeck coefficient and electrical conductivity measurements. Barrier layer alloys were prepared from powder blends of Co (99.999%, 300 mesh), Fe (99.99%, 300 mesh), and Ni (99.99%, 300 mesh). Powders were mixed according to the target compositions of Co_25_Fe_55_Ni_20_, Co_25_Fe_50_Ni_25_, and Co_25_Fe_45_Ni_30_, and subsequently subjected to mechanical alloying via high‐energy ball milling (8000D, SPEX SamplePrep, USA) at a vibration frequency of 10 Hz for 10 h. The weight ratio of ball to material was maintained at 10:1.

### 2.2 Joints and Device Fabrication

Joints were fabricated using a two‐step sintering method. First, the barrier layer was densified by SPS at 1373 K and 100 MPa. It was then co‐sintered with the HH TE material at 1173 K and 65 MPa. The TE module with the barrier layer was machined into dimensions of 3.45 × 3.45 × 4.4 mm^3^, and then placed between two copper electrodes for efficiency testing.

### 2.3 Microstructure Characterization

Phase compositions were characterized by X‐ray diffraction (XRD, Empyrean, PANalytical, Netherlands) operated at 40 kV and 40 mA. Microstructures of the barrier material, HH TE material, and the joint were examined using scanning electron microscopy (SEM, 7900, Hitachi, Japan) and electron probe micro‐analyzer (EPMA, JXA‐8530F PLUS, JEOL, Japan). Scanning transmission electron microscope (STEM, FEI Titan G2 60–300, FEI, USA) samples were prepared by focused ion beam (FIB, LYRA3 FIB‐SEM, TESCAN, Czech Republic) system. Atomic resolution high‐angle annular dark‐field (HAADF) imaging was conducted using a probe‐corrected STEM with a cold field emission source.

### 2.4 TE Properties Test

Electrical conductivity (*σ*) and Seebeck coefficient (*S*) were measured by commercial instruments (LSR‐3, Linseis, Germany) from 323 to 923 K. Thermal diffusivity and density were determined by the flash diffusivity method (LFA457, Netzsch, Germany) and Archimedes’ method, respectively. CTE was measured by a thermal dilatometer (DIL 402SE, Netzsch, Germany). Bonding strength was tested with a material testing machine (Materials Testing5567A, Instron, USA). The contact resistivity at the joint interface was measured via a custom four‐probe system and precisely determined based on the number of jump points at the slope inflection, corresponding to the thickness of the diffusion layer. Device performance was evaluated using a self‐made TE power generation test system.

### 2.5 Calculation Details

E*_IR_* was defined as the formation enthalpy determined from the chemical reaction:

(1) $${\mathrm{Hf}}_{0.6}{\mathrm{Zr}}_{0.4}{\mathrm{NiSn}}_{0.99}+xM\to {\mathrm{Hf}}_{0.6}{\mathrm{Zr}}_{0.4}\mathrm{Ni}+M_{x}{\mathrm{Sn}}_{y}$$

where M*_x_* Sn*_y_* represents Sn‐rich compound in M‐Sn system stabilized at 923–1173 K based on the Open Quantum Materials Database (OQMD) and FactSage Database. CTE data was obtained from MatWeb Material Property Data.

## 3 Results and Discussion

### 3.1 Design Rules for Barrier Layer in Hf_0.6_Zr_0.4_NiSn_0.99_Sb_0.01_

Hf_0.6_Zr_0.4_NiSn_0.99_Sb_0.01_ is selected as the N‐type TE material due to its excellent performance. XRD (Figure S1a) and EPMA (Figure S1b) results confirmed high sintering quality. The material exhibits excellent electrical and thermal properties, with an average *zT* of 0.62 at 923 K (Figure S2), calculated using the temperature‐integration method as defined in Equation (2):

(2) $$zT_{\mathrm{avg}}=\frac{1}{T_{h}-T_{c}}\;\underset{T_{c}}{\int^{T_{h}}}\mathrm{zT}\left(T\right)\mathrm{dT}$$

where 𝑇_𝑐_ = 323 K (room temperature) and 𝑇_ℎ_ = 923 K (maximum testing temperature). The specific calculation entails integrating the *zT* values over the temperature interval of 323–923 K, as depicted in Figure S2e, and subsequently dividing the obtained result by the width of this temperature interval. The interfacial reaction at the heterogeneous interface is widely recognized as a key factor affecting both interface stability and bonding strength. A moderate interfacial reaction can maximize interfacial bonding strength while preserving stability [46, 47]. A single‐element barrier layer often struggles to enhance bonding strength due to limited interface reaction. Therefore, a comprehensive design of multi‐component barrier layer alloys, based on chemical reaction and CTE matching, can effectively address this issue.

For Hf_0.6_Zr_0.4_NiSn_0.99_Sb_0.01_ TE material, Hf and Zr exhibit low diffusion coefficients, while Sb is present in minor amounts; therefore, these elements are not active in promoting interfacial reaction. In contrast, Sn has the lowest melting point (505 K) in this material, weak metal bonding, and a low activation energy (160 kJ/mol). Consequently, Sn demonstrates the highest tendency for rapid diffusion during high‐temperature sintering and will react with the barrier layer to form intermetallic compounds [48, 49]. Furthermore, identifying elements that possess a moderate negative formation energy for reactions with Sn can afford the thermodynamic driving force for forming such intermetallics [47, 49, 50].

The fundamental criteria for screening barrier layer materials include high thermal conductivity, high electrical conductivity, and high melting point [46, 47]. Thus, metals M (where M = Ag, Cu, Fe, Ni, Co, Ti, W, Zr, Nb, Ta, Mo, and Cr) are considered as potential candidates. The main reactions between these metals and the TE material can be described as Equation (1). Table 1 summarizes the main interfacial reactions between these candidate metals and Sn during sintering at 923–1173 K, along with the interface reaction energy (E*_IR_*) of the resulting intermetallic compounds, as determined by their thermodynamic stability [51, 52, 53, 54, 55, 56, 57].

**TABLE 1:** CTEs of candidate elements and their main interfacial reaction with Sn at 923–1173 K.

Metal	Main reaction	Interfacial product compounds	E*_IR_* (eV/atom)	CTE (10^−6^ K^−1^)
Ag	3Ag+Sn→Ag_3_Sn	Ag_3_Sn	−0.05	19.6
Cu	6Cu+5Sn→Cu_6_Sn_5_	Cu_6_Sn_5_	−0.061	16.4
Fe	Fe+Sn→FeSn	FeSn	−0.057	12.2
Ni	3Ni+2Sn→Ni_3_Sn_2_	Ni_3_Sn_2_	−0.313	13.1
Co	Co+Sn→CoSn	CoSn	−0.162	12.5
Ti	5Ti+3Sn→Ti_5_Sn_3_	Ti_5_Sn_3_	−0.417	8.9
W	—	—	—	4.4
Zr	5Zr+3Sn→Zr_5_Sn_3_	Zr_5_Sn_3_	−0.6	5.8
V	—	—	—	8.33
Nb	3Nb+Sn→Nb_3_Sn	Nb_3_Sn	−0.195	7
Mo	—	—	—	5.35
Cr	—	—	—	6.2

Ni is initially selected as a candidate due to its moderate E*_IR_*, which avoids excessive interfacial reactions while ensuring sufficient bonding strength. Ni also exhibits excellent chemical compatibility with the Hf_0.6_Zr_0.4_NiSn_0.99_Sb_0.01_ TE material. Regarding CTE, previous studies report values of 8.6 × 10^−6^ K^−1^ for ZrNiSn and 8.38 × 10^−6^ K^−1^ for HfNiSn [58]. Based on the design concept of multi‐component alloys and CTE matching, Fe and Co elements with relatively lower E*_IR_* are chosen as auxiliary components to modulate interfacial reaction behavior. Among the intermetallic compounds FeSn, Ni_3_Sn_2_, and CoSn, CoSn is a brittle phase [59], necessitating strict control of Co content at low levels in the alloy.

Based on the above analysis, we regulate the interfacial reaction by adjusting the ratio between Ni (with a relatively higher E*_IR_*) and Fe (with a relatively lower E*_IR_*), while exploring the influence of varying reaction degrees on joint performance. Three alloys with various Ni/Fe ratios are designed, which are Co_25_Fe_55_Ni_20_, Co_25_Fe_50_Ni_25_, and Co_25_Fe_45_Ni_30_ alloys (hereinafter referred to as Ni_20_, Ni_25_, and Ni_30_).

### 3.2 Interface Microstructure and Performance

**FIGURE 2:**
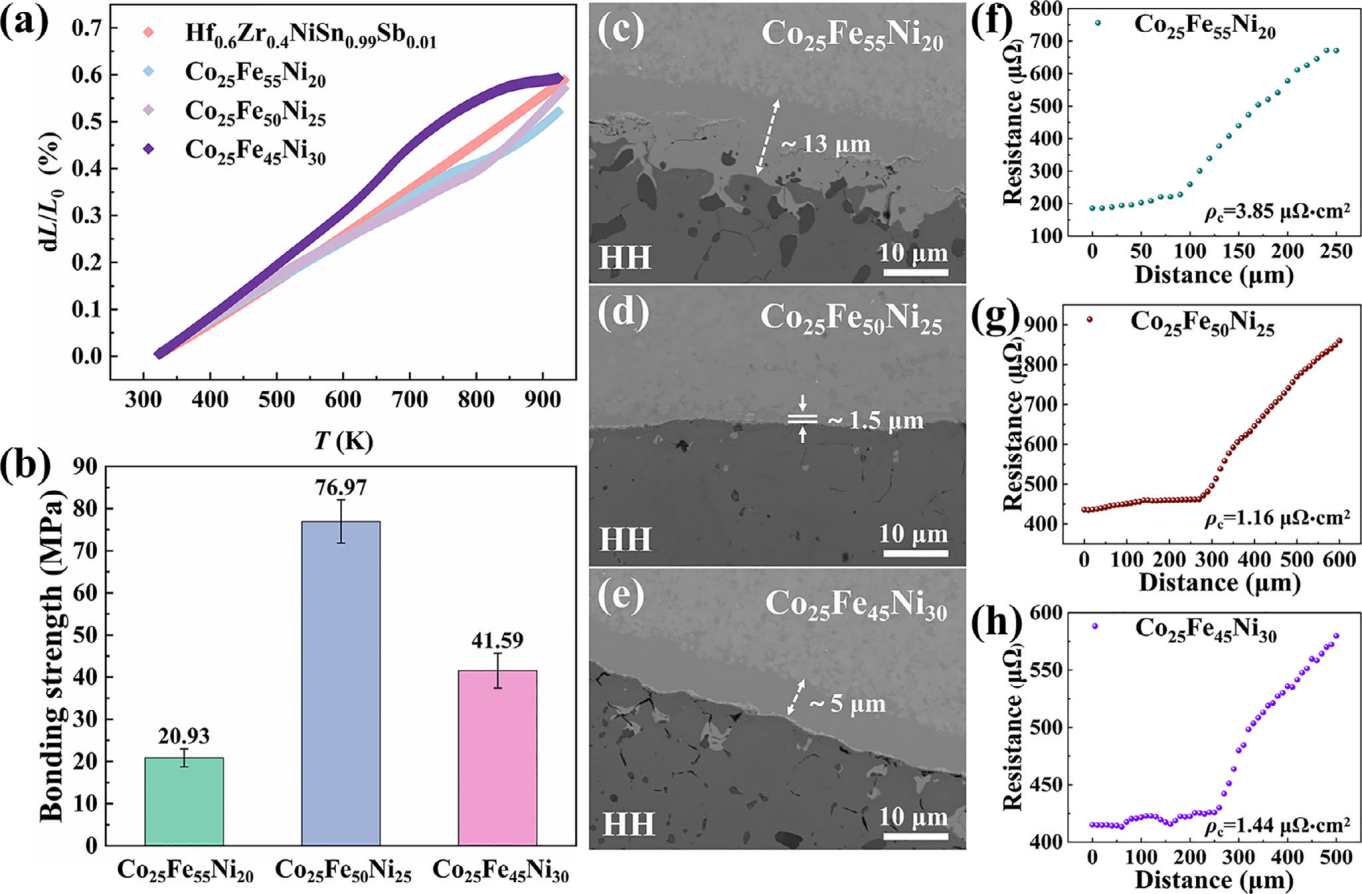
Design of Co‐Fe‐Ni barrier layer: (a) thermal expansion d*L*/*L_0_* of the Co‐Fe‐Ni barrier layers as a function of temperature, (b) interfacial bonding strength of welded joints with different Fe/Ni ratios, BSE images of welded joints for (c) Ni_20_, (d) Ni_25_, and (e) Ni_30_, complete contact resistivity results for (f) Ni_20_, (g) Ni_25_, and (h) Ni_30._

XRD characterization reveals that the Ni_20_, Ni_25_, and Ni_30_ alloys all exhibit a single FCC phase (Figure S3a), confirming that Co, Fe, and Ni were fully mechanical alloyed [60]. EPMA results show that a homogeneous microstructure, a dense and defect‐free surface, and uniform element distribution, attesting to excellent sintering quality (Figure S3b–e). Figure 2a illustrates the functional relationship between the thermal expansion (d*L*/*L_0_*) and temperature, revealing that all three Co‐Fe‐Ni barrier layers exhibit excellent thermal expansion matching with HH. At 923 K, the difference in d*L*/*L_0_* between HH and Co_25_Fe_55_Ni_20_, Co_25_Fe_50_Ni_25_, and Co_25_Fe_45_Ni_30_ are 12%, 3%, and 2%, respectively. These values are consistent with predictions and confirm the feasibility of the joints.

The SPS‐sintering joints achieve tight, crack‐free bonding, attributable to the excellent thermal expansion matching between the Co‐Fe‐Ni barrier layers and HH (Figure 2c–e). All three joints exhibit a uniform and continuous diffusion layer at the interface. Among them, the Ni_25_ barrier layer displays the shallowest diffusion depth of only 1.5 µm. Figure 2b shows that the Ni_25_ barrier layer achieves the maximum interfacial bonding strength of 77.0 MPa, far exceeding the qualified threshold of 20 MPa [46, 47]. With an increasing Ni/Fe ratio, the interfacial bond strength initially increases and then decreases (the specific test curve is shown in Figure S5). The Ni_25_ barrier layer interface also exhibits the lowest contact resistivity of 1.16 µΩ·cm^2^ (Figure 2f–h). The nonlinear behavior characterized by two distinct changes in slope, as observed in Figure 2g,h, arises from the formation of a seepage zone resulting from the infiltration of intermetallic compounds into the TE material through the diffusion layer (Figure S8). The contact resistivity at the joint can be considered negligible, as the resistivity of the seepage zone is comparable to that of the TE material. However, the higher resistivity of the intermetallic compound within the percolation region relative to the TE material indicates that the infiltration of intermetallic compounds influences the TE transport properties in this region. The observed rise in resistivity demonstrates a reduction in carrier concentration and an accompanying increase in the Seebeck coefficient, as described by Equations (3) and (4) [61]. The decrease in electrical conductivity, combined with the scattering of second‐order relative phonons induced by the infiltration of intermetallic compounds, contributes to a reduction in thermal conductivity, as expressed in Equations (5) and (6) [20].

(3) $$\rho =\frac{1}{\mathrm{ne}\mu }\;$$

(4) $$S\;=\frac{8\pi^{2}k_{B}^{2}}{3eh^{2}}\;m^{*}T{\left(\frac{\pi }{3n}\right)}^{2}$$

(5) $$\kappa_{e}\;=\;L\sigma T$$

(6) $$\kappa \;=\;\kappa_{e}+\kappa_{l}$$

here, *ρ* represents the resistivity, *n* denotes the carrier concentration, and *μ* signifies the carrier mobility. *S* stands for the Seebeck coefficient, *k_B_* represents the Boltzmann constant, *h* denotes Planck's constant, *m* ^∗^ represents the effective mass of carriers, and *T* represents the absolute temperature. *κ* denotes the total thermal conductivity, *κ_e_* represents the electronic thermal conductivity, *L* represents the Lorentz constant, *σ* represents the electrical conductivity, and *κ_l_* represents the lattice thermal conductivity. Based on diffusion depth, bonding strength, and contact resistivity, the Ni_25_ barrier layer interface promotes the most favorable interface reaction.

**FIGURE 3:**
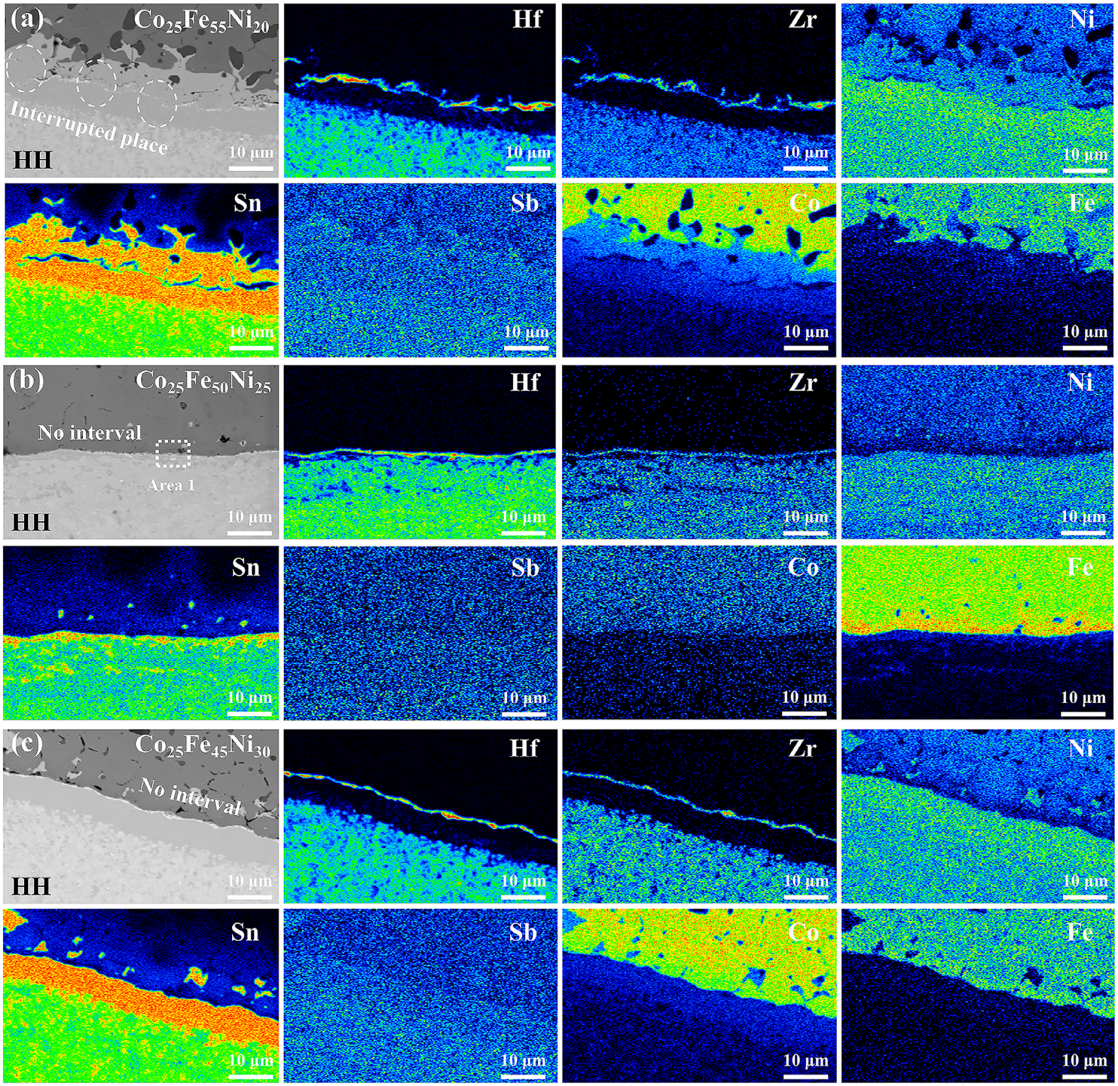
Microstructure and corresponding elemental mappings of Hf, Zr, Ni, Sn, Sb, Co, and Fe at different joints: (a) HH/Ni_20_, (b) HH/Ni_25_, and (c) HH/Ni_30._

EPMA is used to analyze the favorable interface reaction at the Ni_25_ barrier layer and the effect of varying Ni/Fe ratios. The interface behavior of each element is shown in Figure 3. Sn is clearly identified as the main enrichment element in the diffusion layer due to its low melting point (505 K), weak metal bonding, and low activation energy (160 kJ/mol). These properties enable Sn to diffuse rapidly and readily react with the barrier layer during high‐temperature sintering, forming intermetallic compounds [48, 49], consistent with prior prediction.

Notably, a Hf‐Zr enrichment layer approximately 0.5 µm thick is observed at the barrier layer boundary. As the Ni/Fe ratio increases, this layer transits from a discontinuous to a dense and continuous morphology. At the Ni_20_ joint interface (Figure 3a), significant amounts of Sn diffuse into the barrier layer through gaps in the Hf‐Zr layer. In contrast, only trace amounts of Sn diffuse through the Hf‐Zr layer at the Ni_25_ and Ni_30_ joint interfaces (Figure 3b,c). This confirms that the Hf‐Zr enrichment layer acts as a chemical barrier, effectively inhibiting interdiffusion and blocking Sn from diffusing from the reaction layer into the barrier layer. We conclude that increasing the Ni/Fe ratio facilitates the formation of a dense and continuous Hf‐Zr chemical barrier. To verify this, a Co_25_Fe_60_Ni_15_ barrier alloy with a lower Ni/Fe ratio is fabricated (Figure S4). Its Hf‐Zr layer is markedly more discontinuous than that of the Ni_20_ joint, allowing substantial Sn diffusion. Thus, a higher Ni/Fe ratio promotes the formation of a dense, continuous Hf‐Zr enrichment layer that acts as an effective chemical barrier against Sn diffusion.

**FIGURE 4:**
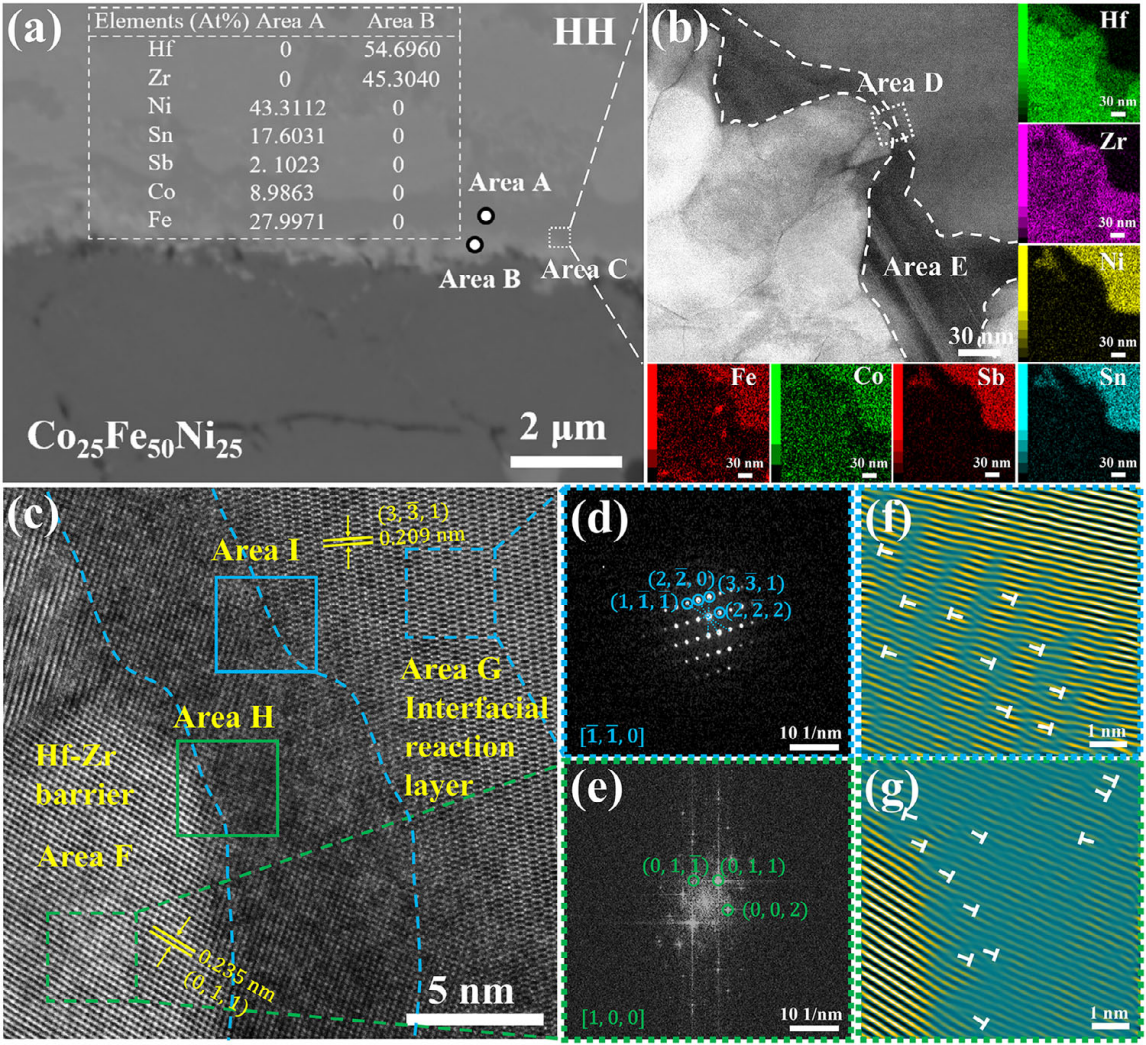
Microstructures of the as‐fabricated HH/Ni_25_ interface: (a) microstructure and element point distribution, (b) HAADF image and corresponding elemental mapping, (c) interface lattice matching between the Hf‐Zr chemical barrier and the reaction layer, (d) FFT images corresponding to the reaction layer, (e) Fourier transformation images corresponding to Hf‐Zr chemical barrier, and (f, g) corresponding IFFT image showing the interface featuring a high‐density edge dislocation region.

Elemental point analysis is performed on a locally magnified area (Area 1) at the Ni_25_ joint interface (Figure 3b) to determine the composition of the diffusion and the Hf‐Zr enrichment layers (Figure 4a). Results from Area A indicate that Co, Fe, and Ni all react with Sn within the diffusion layer, forming various intermetallic compounds containing these elements. Sb content is negligible due to its low concentration. Ni exhibits the highest atomic percentage, indicating that Ni_3_Sn_2_ is the dominant phase, consistent with the design principle of a larger E*_IR_* (−0.313 eV/atom) between Ni and Sn. Combined with the bonding strength results (Figure 2b), the formation of an appropriate amount of Ni_3_Sn_2_ is conducive to maintaining high bonding strength, as observed at the Ni_25_ joint interface.

Element point distribution results from Area B verify that the Hf‐Zr chemical barrier is essentially a Hf‐Zr binary intermetallic compound, with no other elements participating in its formation. During high‐temperature sintering, interfacial elements, including Ni, Sn, Sb, Co, and Fe, react under binding energy driving forces to form a reaction layer. Hf and Zr are transition metals with high melting points, large atomic radii, and extremely low solid‐state self‐diffusion coefficients [62, 63]. Specifically, their self‐diffusion coefficients are usually 5–8 orders of magnitude smaller than those of Ni, Co, Fe, and Sn [48, 64, 65]. Consequently, Hf and Zr exhibit negligible migration within the reaction layer; their in situ retention leads to locally elevated concentrations, forming the Hf‐Zr chemical barrier. The timely formation of a dense and continuous Hf‐Zr chemical barrier hinders further Sn diffusion and suppresses ongoing reactions with Co, Fe, and Ni, thereby ensuring controlled formation of Ni_3_Sn_2_ and moderated interface reaction progression. Elemental distribution results (Figure 4a) further verify that the discontinuity of the Hf‐Zr barrier at low Ni/Fe ratios stems from a limited interfacial reaction extent, resulting in insufficient in situ accumulation of Hf and Zr.

To explore the mechanism by which the Hf‐Zr barrier inhibits Sn diffusion, STEM analysis is performed on typical coexisting regions of the Hf‐Zr barrier and the interfacial reaction layer (Area C in Figure 4a). Figure 4b shows three regions with distinct contrast. Nano‐scale elemental mapping revealed that Zr enrichment in Area E, with the Hf‐Zr barrier exhibiting a core‐shell structure, where a Zr‐rich core is encapsulated by a Hf‐rich layer. Except for trace Fe and Co precipitates, the distribution of other elements is consistent with that of element points in Figure 4a. Sn is completely confined outside the Hf‐Zr barrier. Subsequently, Area D, encompassing both the barrier and reaction layer, is selected for atomic‐scale characterization (Figure 4c). High‐resolution STEM images further confirm direct atomic bonding between adjacent phases. The lattice spacing of the [100] oriented Hf‐Zr barrier surface (Area F) is measured as 0.235 nm, while that of the [$\bar{1}\bar{1}$0] oriented reaction layer (Area G) is 0.209 nm, yielding a mismatch of approximately 11%. IFFT images of Area H and I (Figure 4f,g) reveal a high‐density edge dislocation region between the Hf‐Zr barrier and the reaction layer, arising from lattice mismatch [66, 67]. This dislocation region acts as a robust diffusion barrier, significantly suppressing cross‐interface atomic diffusion [66]. The atomic‐scale coherent interface structure in Figure S6a–c confirms that there is no diffusion barrier at the interface between the reaction layer and the HH. Based on the above analysis, the inhibition of Sn diffusion mainly depends on the Hf‐Zr barrier side.

**FIGURE 5:**
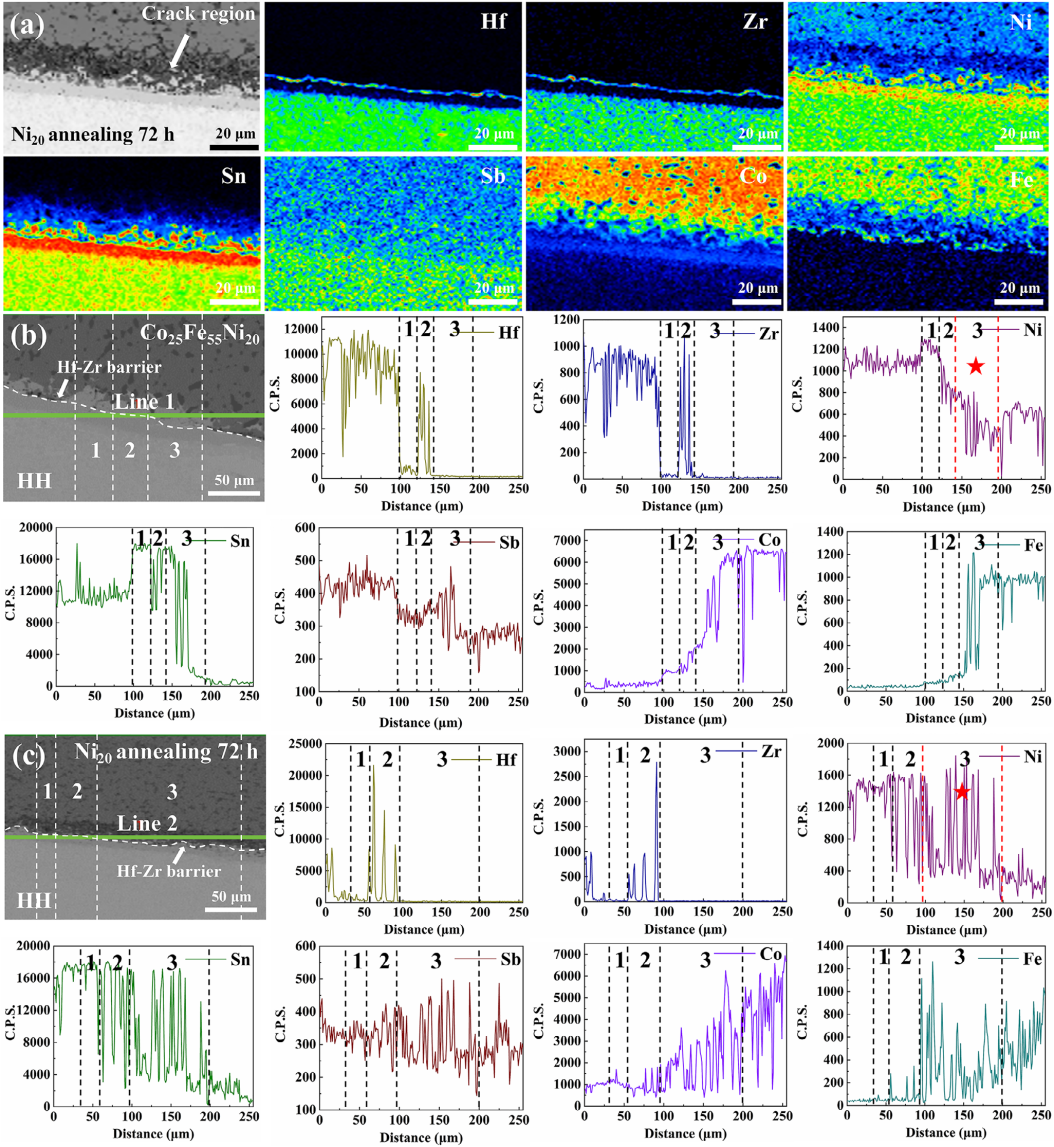
Element distribution at different joints: (a) interface morphology and element distribution of the Ni_20_/HH joint after annealing for 72 h, element line distributions of the Ni_20_/HH joint (b) before annealing, and (c) after annealing.

To verify the effect of Sn diffusion, induced by the discontinuity, on joint thermal stability, the Ni_20_ joint is annealed at 973 K for 72 h (Figure 5a). Numerous cracks develop in the barrier layer regions where Sn has diffused prior to annealing. Elemental line scans across three regions (diffusion layer: Region 1; Hf‐Zr barrier: Region 2; Sn‐diffused barrier region: Region 3) are analyzed before and after annealing (Figure 5b,c). The distributions of Hf, Zr, Sn, Sb, Co, and Fe remain consistent. In contrast, the Ni content in Region 3 (i.e., the crack region) increases significantly after annealing. This is attributed to the vigorous reaction between the diffused Sn and Ni within the barrier layer during annealing, forming a large quantity of Ni_3_Sn_2_ intermetallic compounds [48, 64].

Therefore, the brittle fracture mechanism can be summarized as follows: Owing to the discontinuity of the Hf‐Zr chemical barrier, Sn diffuses into the barrier layer; during high‐temperature annealing, Sn reacts vigorously with the abundant Ni within the barrier alloy, forming excessive Ni_3_Sn_2_ [68]. This aligns with the bonding strength (Figure 3b), indicating that an appropriate amount of Ni_3_Sn_2_ contributes to ensuring high interfacial bonding strength, whereas excessive formation promotes brittleness.

**FIGURE 6:**
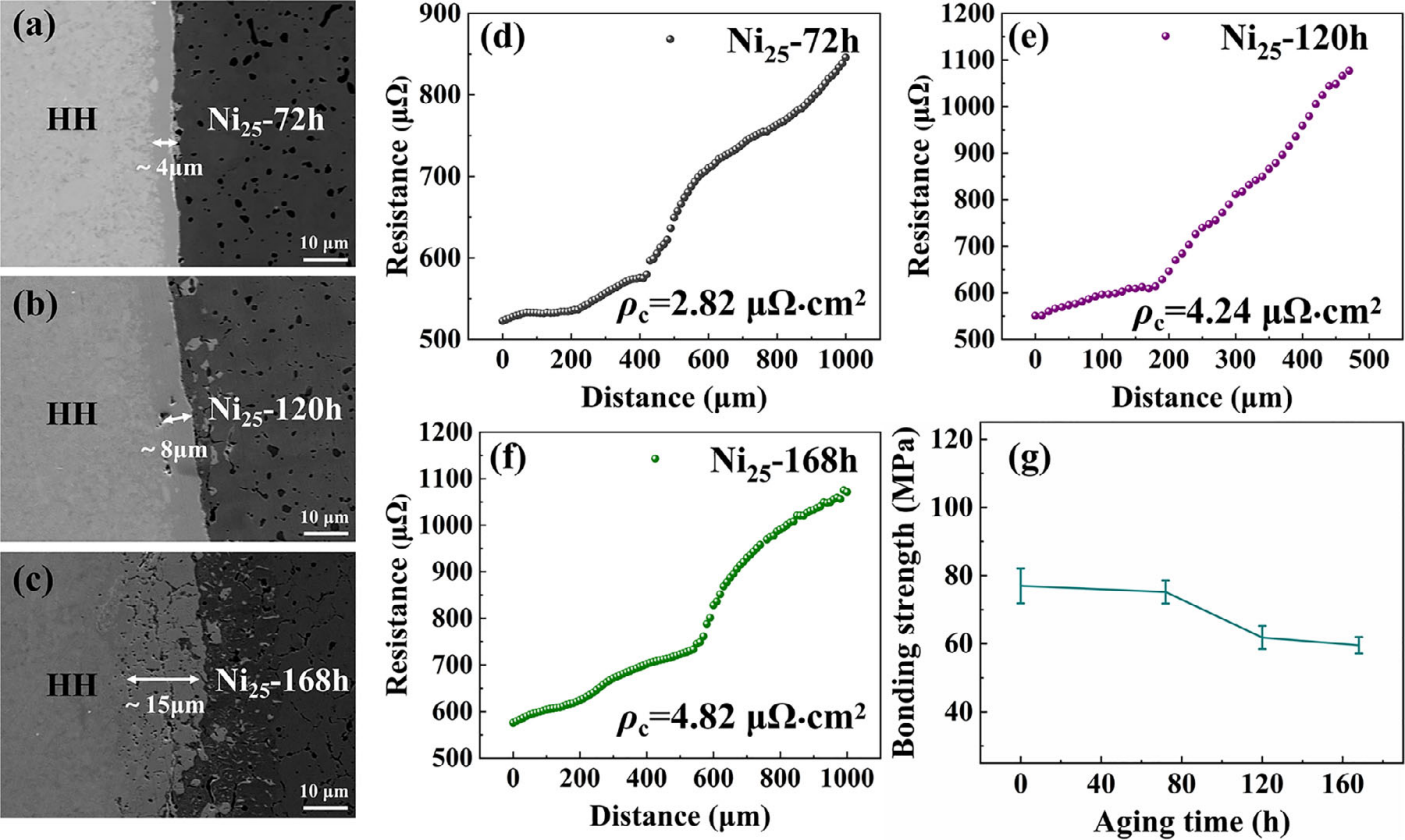
Microstructure of the Ni_25_/HH joint after different annealing times: (a) 72 h, (b) 120 h, and (c) 168 h; contact resistivity after (d) 72 h, (e) 120 h, (f) 168 h, and (g) bonding strength after different annealing times.

In summary, moderate interfacial reaction is key to achieving high bonding strength, while a dense and continuous Hf‐Zr chemical barrier guarantees excellent thermal stability. Based on a comprehensive evaluation of diffusion depth, contact resistivity, and bonding strength, the Ni_25_ alloy is selected as the optimal barrier for the Hf_0.6_Zr_0.4_NiSn_0.99_Sb_0.01_ TE material. To further evaluate its thermal stability, Ni_25_ joints are annealed at 923 K for 72, 120, and 168 h, respectively (Figure 6). The interface remains densely bonded without obvious cracks throughout annealing. After annealing for 168 h, the diffusion layer thickness is only 15 µm. After annealing for 168 h, the contact resistivity increases slightly to 4.82 µΩ·cm^2^, which remains within the acceptable range for device service [46, 47]. This behavior is related to the local redistribution of the Sn element near the interface and the trace oxygen adsorption at the interface. Nonlinear fitting results indicate that the contact resistivity remains consistently below 5 µΩ·cm^2^ throughout the aging process, provided that no interfacial fracture occurs, as demonstrated in Figure S7 [50]. The nonlinear behaviors (exhibiting two slope changes) observed in Figure 6d,f arise from the same underlying causes and effects as those described for Figure 2g,h. The joint sustains high bonding strength during annealing, retaining 60.0 MPa after 168 h, with an attenuation of only 22.08%.

### 3.3 Single‐Leg Device Performance

**FIGURE 7:**
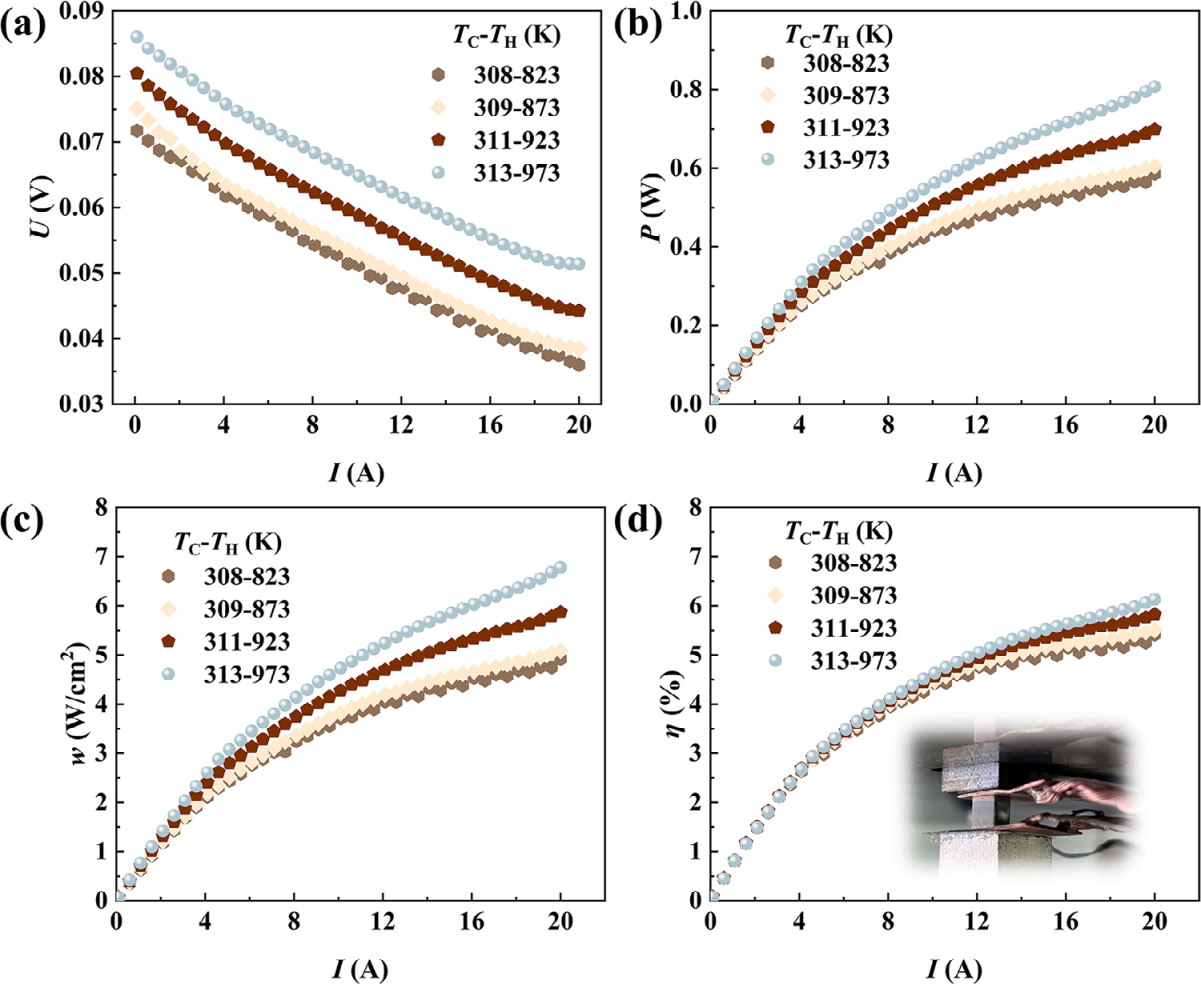
Output performance of the single‐leg N‐type TE device: (a) output voltage, (b) output power, (c) output power density, and (d) conversion efficiency.

The applicability of the developed Co_25_Fe_50_Ni_25_ barrier layer is validated by fabricating an N‐type single‐leg device with dimensions of 3.45 × 3.45 × 4.4 mm^3^, as shown in Figure 7d. A self‐made experimental setup is employed to investigate the correlation between device performance and operating current at different hot‐end temperatures (Figure 7a–d). The *U*–*I* curve (Figure 7a) exhibits an excellent linear relationship. Output power increases with increasing temperature difference (Figure 7b), reaching 0.807 W at a current of 20 A and a temperature difference of 660 K, with a continuing upward trend.

As shown in Figure 7c,d, at a temperature difference of 660 K, the device achieves a power density exceeding 6.78 W/cm^2^ and a conversion efficiency higher than 6.13%, both highly competitive for single‐leg devices. At elevated temperature, increased interface thermal resistance between the upper surface of the device and the heat source, along with enhanced convective heat transfer and thermal radiation at the cold‐ and hot‐ends, reduces the actual temperature difference below the set value. These factors, particularly prominent in single‐leg devices, account for the measured conversion efficiency being lower than the theoretical value [40, 42].

## 4 Conclusions

Guided by the principle that a moderate interfacial reaction promotes atomic bonding between the barrier layer and TE materials, thereby significantly enhancing bonding strength. A CoFeNi barrier layer alloy is comprehensively screened and determined based on the thermodynamic parameters. The Co_25_Fe_50_Ni_25_ alloy exhibits optimal interfacial bonding performance. Building upon this controlled interfacial reaction, a dense and continuous Hf‐Zr chemical barrier is constructed to prevent joint degradation. This design yields a higher bonding strength of 77.0 MPa and a low contact resistivity of 1.16 µΩ·cm^2^. Even after annealing at 923 K for 168 h, the bonding strength remains as high as 60.0 MPa, with the contact resistivity below 5 µΩ·cm^2^. Finally, a single‐leg N‐type HH device fabricated using the Co_25_Fe_50_Ni_25_ barrier alloy achieves a high output power density exceeding 6.78 W/cm^2^ and a conversion efficiency over 6.13% at elevated temperatures. This study provides an effective barrier layer design paradigm for improving interfacial bonding strength and ensuring high structural stability in high‐temperature TE devices.

## Conflicts of Interest

The authors declare no conflict of interest.

## Supporting information




**Supporting File**: advs74870‐sup‐0001‐SuppMat.docx.

## Data Availability

The data that support the findings of this study are available from the corresponding author upon reasonable request.
