# Supplementary material for: Controlled Moderate Interfacial Reaction Facilitates High‐Strength Low‐Resistivity Barrier Layer for Half‐Heusler Thermoelectrics
Source: Adv Sci (Weinh). 2026 Mar 17;13(31):e74870. doi: 10.1002/advs.74870 (PMC13588045; doi:10.1002/advs.74870)
Supplement: Supplementary file 1 [file ADVS-13-e74870-s001.docx]

*Supporting Information*

**Controlled Moderate Interfacial Reaction Facilitates High-Strength Low-Resistivity Barrier Layer for Half-Heusler Thermoelectrics**

Jian Liang^1^, Shuyue Tan^1^, Lifeng Jiang^1^, Wen Zhang^1^, Hongda Song^1^, Xinghui Wang^1^, Haiping Guo^1^, Huijun Kang^1, 2*^, Rongchun Chen^1*^, Enyu Guo^1, 2^, Zongning Chen^1, 2^, Tongmin Wang^1, 2*^

^1^ Key Laboratory of Solidification Control and Digital Preparation Technology (Liaoning Province), School of Materials Science and Engineering, Dalian University of Technology, Dalian, 116024, China

^2^ Ningbo Institute of Dalian University of Technology, Ningbo 315000, China

* Corresponding authors.

E–mail addresses: kanghuijun@dlut.edu.cn (H. J. Kang); crc618@dlut.edu.cn (R. C. Chen); tmwang@dlut.edu.cn (T. M. Wang)

ORCiD·ID: 0000-0002-7379-7861; 0000-0003-1499-0837; 0000-0001-9871-1166

Figure S1. Phase composition and microstructures of Hf_0.6_Zr_0.4_NiSn_0.99_Sb_0.01_: a) XRD patterns, b) microstructure, and corresponding elemental mappings of c) Hf, d) Zr, e) Ni, f) Sn, and g) Sb

Figure S2. Temperature-dependent thermoelectric properties of Hf_0.6_Zr_0.4_NiSn_0.99_Sb_0.01_: a) *S*, b) *σ*, c) PF, d) *κ*, and e) *zT*

Figure S3. Phase composition and microstructures of Co_25_Fe_55_Ni_20_, Co_25_Fe_50_Ni_25_, and Co_25_Fe_45_Ni_30_ alloys: a) XRD patterns, b) microstructure of Co_25_Fe_50_Ni_25_, and corresponding elemental mappings of c) Co, d) Fe, and e) Ni

Figure S4. Microstructure and corresponding elemental mappings of Co_25_Fe_60_Ni_15_/HH joint

Figure S5. Interfacial bonding strength of welded joints with different Fe/Ni ratios: a) Co_25_Fe_55_Ni_20_, b) Co_25_Fe_55_Ni_20_, and c) Co_25_Fe_55_Ni_20_

Figure S6. Microstructures of the as-fabricated HH/Ni_25_ interface: a) microstructure, b) HAADF image and corresponding elemental mapping of Area a, and c) atomic-scale imaging of Area b

Figure S7. The contact resistivity as a function of the aging time for Ni_25_/HH joint

Figure S8. Microstructure and corresponding elemental mappings of Co_25_Fe_45_Ni_30_/HH joint
